# Hydrogen-Rich Water Attenuates Diarrhea in Weaned Piglets via Oxidative Stress Alleviation

**DOI:** 10.3390/biology14080997

**Published:** 2025-08-05

**Authors:** Pengfei Zhang, Jingyu Yang, Zhuoda Lu, Qianxi Liang, Xing Yang, Junchao Wang, Jinbiao Guo, Yunxiang Zhao

**Affiliations:** 1 College of Animal Science and Technology, Guangxi University, Nanning 530004, China; pengfeizhang2016@163.com (P.Z.); heyimzhuodalu@163.com (Z.L.); liangqx314@163.com (Q.L.); yxing202303@163.com (X.Y.); 16635424929@163.com (J.W.); 2 School of Life Sciences and Engineering, Foshan University, Foshan 528231, China; yangjingyu1188@foxmail.com

**Keywords:** hydrogen-rich water, weaned piglets, growth performance, oxidative stress, diarrhea, gut microbiota, hepatic metabolism

## Abstract

Early weaning of piglets elicits weaning stress, which in turn induces oxidative stress and consequently impairs growth and development. Hydrogen-rich water (HRW), characterized by selective antioxidant properties, mitigates oxidative stress damage and serves as an ideal intervention. This study aimed to evaluate the effects of HRW on weaned piglets, specifically investigating its impact on growth performance, diarrhea incidence, antioxidant function, intestinal morphology, gut microbiota, and hepatic metabolites. The results demonstrate that HRW significantly increased the average daily feed intake and significantly reduced the diarrhea rate in weaned piglets. Analysis of serum oxidative stress indicators revealed that HRW significantly elevated the activities of total antioxidant capacity and total superoxide dismutase while significantly decreasing malondialdehyde concentration. Assessment of intestinal morphology showed that HRW significantly increased the villus height to crypt depth ratio in the duodenum, jejunum, and ileum. Microbial analysis indicated that HRW significantly increased the abundance of *Prevotella* in the colon. Furthermore, HRW increased the abundance of beneficial bacteria, such as *Akkermansia*, in the jejunum and cecum, while concurrently reducing the abundance of harmful bacteria like *Escherichia*. Hepatic metabolite profiling revealed that HRW significantly altered the metabolite composition in the liver of weaned piglets. Differentially abundant metabolites were enriched in oxidative stress-related KEGG pathways, including ABC transporters; pyruvate metabolism; autophagy; FoxO signaling pathway; glutathione metabolism; ferroptosis; and AMPK signaling pathways. In conclusion, HRW alleviates diarrhea and promotes growth in weaned piglets by enhancing antioxidant capacity. These findings provide a scientific foundation for the application of HRW in swine production and serve as a reference for further exploration into the mechanisms underlying HRW’s effects on animal health and productivity.

## Simple Summary

Early weaning in piglets induces weaning stress, leading to intracellular reactive oxygen species accumulation. This subsequently triggers oxidative stress and causes intestinal mucosal damage. Currently, there remains no effective solution to this problem. Hydrogen-rich water, with its selective antioxidant properties, mitigates oxidative stress damage and represents a promising antioxidant intervention. This study substituted drinking water with hydrogen-rich water in weaned piglets to investigate its effects on growth performance, serum antioxidant capacity, intestinal morphology, gut microbiota composition, and hepatic metabolism. Key findings demonstrate that hydrogen-rich water reduces diarrhea incidence by alleviating oxidative stress. This work establishes a scientific foundation for utilizing hydrogen-rich water as a residue-free, eco-friendly antioxidant in commercial swine operations.

## 1. Introduction

Early weaning is a widely adopted practice in commercial pig farming [1]. However, the abrupt dietary transition post-weaning imposes severe stress on piglets, resulting in weaning stress syndrome. This syndrome is characterized by elevated intracellular ROS levels and subsequent intestinal mucosal damage [2]. Studies have shown that the transition from liquid to solid feed after weaning leads to decreased feed intake and body weight gain, along with an increased incidence of diarrhea in piglets [3]. Consequently, mitigating weaning stress in piglets has become a crucial challenge requiring solutions in modern swine production.

Hydrogen-enriched water (HRW), as a novel functional water, demonstrates distinctive bio-efficacy in healthcare applications mediated by molecular hydrogen (H_2_). H_2_ exhibits highly selective antioxidant capacity. Its small molecular size allows it to readily penetrate cell membranes and react with intracellular ROS [4]. H_2_ exhibits mild reducibility and does not interfere with normal physiological redox reactions within the organism [5]. Research indicates that H_2_ can selectively scavenge hydroxyl radicals and peroxynitrite anions, thereby alleviating oxidative stress damage caused by ischemia–reperfusion in organs such as the liver, heart, and intestine, ultimately protecting cells [6]. H_2_ also exerts anti-apoptotic effects by inhibiting Caspase-1 activity, modulates pro-inflammatory cytokines, and suppresses acute lung injury induced by traumatic brain injury [7]. It is recognized for its antioxidant, anti-inflammatory, and anti-apoptotic properties [8]. Although preliminary results from clinical trials and studies suggest the efficacy of HRW, further research is needed to substantiate these findings.

This study aims to systematically evaluate the effects of HRW on the growth performance, intestinal health, and hepatic metabolism of weaned piglets. The findings are expected to provide a scientific basis for the application of HRW as a novel, residue-free, and environmentally friendly green antioxidant in swine production.

## 2. Materials and Methods

### 2.1. Experimental Animals and Grouping

A total of sixty 21-day-old weaned castrated male piglets (Landrace × Large White crossbred) (Shanxi Jiabai Breeding Pig Breeding Co., Ltd., Lvliang, China) were selected for the experiment. The initial body weight ranged from 6.42 ± 0.85 kg (mean ± SD), with no significant intergroup difference in body weight (*p* > 0.05) among individuals. The piglets were randomly assigned to one of two dietary treatment groups (n = 6 replicates per group, with 5 pigs per replicate): Control group (C): Received purified water from a filtered water purification system; Hydrogen-rich water group (H): Received HRW at a concentration of 2000 ppb parts per billion throughout the experiment. The experimental period lasted for 14 days. Hydrogen-enriched water for the experimental groups was supplied using a custom-built hydrogen water generator (Foshan Jinming Environmental Technology Co., Ltd., Foshan, China). Tap water intended for pigs was first filtered through a purification system. Hydrogen gas was then produced via an electrolysis module and dissolved into the purified water under high pressure using a pressurization pump. The hydrogen-enriched water was subsequently stored in a pressurized tank and delivered to the piglet drinking nipples. To ensure consistent dissolved hydrogen concentration, the device was designed with a pressure-sensing activation mechanism. This system initiates the production and delivery of hydrogen-enriched water only when piglets initiate drinking, triggered by the pressure drop at the nipple. The dissolved hydrogen concentration in the water dispensed from the nipples in the hydrogen-enriched water experimental group was monitored twice daily.

### 2.2. Animal Management

All experimental pigs were co-housed in an environmentally controlled facility with standardized natural lighting and mechanical ventilation. Throughout the 14-day trial, ambient temperature was maintained at 25 ± 1 °C and relative humidity at 60–70% using automated climate control systems. Pigs were provided ad libitum access to feed with scheduled provision at 08:00 and 16:00 daily.

### 2.3. Growth Performance Measurement

On the first and final day of the experiment, all weaned piglets were weighed individually following a 12 h fast. Daily records were maintained throughout the trial period for feed offered, feed refusal, and diarrhea occurrence. The following parameters were calculated: diarrhea incidence, average daily gain (ADG), average daily feed intake (ADFI), and feed conversion ratio (FCR).

### 2.4. Sample Collection and Processing

On the final day of the experiment, all piglets were fasted for 12 h. Blood samples were then collected from the anterior vena cava of all weaned piglets. Serum was separated from the clotted blood for subsequent analysis of antioxidant capacity indicators. Six piglets per group were randomly selected and euthanized. Segments of the duodenum, jejunum, and ileum were collected for intestinal morphological analysis. Luminal contents from the jejunum, ileum, cecum, colon, and rectum were collected for gut microbiota analysis. Liver tissue samples were collected and immediately snap-frozen in liquid nitrogen for subsequent metabolomic analysis

### 2.5. Assessment of Antioxidant Parameters

Serum antioxidant capacity was evaluated using established biochemical assays. Total antioxidant capacity (T-AOC) was measured by ABTS radical cation decolorization assay, total superoxide dismutase (T-SOD) activity was measured via WST-1 reduction method, and malondialdehyde (MDA) concentration was measured through thiobarbituric acid reactive substance assay. All procedures were performed strictly following manufacturer’s protocols (Jiancheng Bioengineering Institute, Nanjing, China).

### 2.6. Intestinal Morphometric Analysis

Fixed intestinal segments underwent standardized histological processing: 24 h fixation in 4% paraformaldehyde, 2 h running water rinse, ethyl alcohol gradient dehydration, xylene clearing, paraffin embedding, and 5 μm sectioning. Deparaffinization with xylene and rehydration was conducted through ethanol gradient, hematoxylin and eosin (H & E) staining, differentiation in 1% acid–alcohol, bluing in running water, ethanol gradient dehydration, xylene clearing, and neutral balsam mounting. Villus height and crypt depth were measured using Image-Pro Plus, with villus height-to-crypt depth (VH/CD) ratio calculated from intact villi per sample.

### 2.7. Gut Microbiota Profiling

Microbial genomic DNA was extracted from luminal contents using QIAamp Fast DNA Stool Mini Kit (Tiangen Biotech, Beijing, China). The V3-V4 hypervariable region of bacterial 16S rRNA gene was amplified with primers 338F/806R. Purified amplicons were ligated to Illumina adapters for library construction. Paired-end sequencing (2 × 250 bp) was performed on Novaseq 6000 platform (Illumina, San Diego, CA, USA). Bioinformatic processing included Trimmomatic for raw read quality control, FLASH for paired-end read merging, QIIME2 for chimera removal (UCHIME), species annotation via SILVA database (v138) with Naive Bayes classifier, α-diversity indices (Shannon, Simpson) calculated using vegan package in R, and β-diversity assessed by Bray–Curtis distance (PCoA).

### 2.8. Hepatic Metabolomics Profiling

Liver tissues were pulverized in liquid nitrogen, followed by metabolite extraction with 70% methanol aqueous solution containing internal standards. After vortexing and centrifugation, supernatants were collected and incubated at −20 °C for 30 min. Secondary centrifugation yielded clarified supernatants for LC-MS/MS analysis. Chromatographic separation was achieved on a ACQUITY Premier HSS T3 Column (Waters, Milford, CT, USA) with the following: mobile phase A: 0.1% formic acid in water; mobile phase B: 0.1% formic acid in acetonitrile; flow rate: 0.4 mL/min; and gradient program: 0–1 min 5% B, 1–9 min 5–95% B, 9–10 min 95% B, 10–10.1 min 95–5% B, and 10.1–12 min 5% B. Mass spectrometry was performed in positive ion mode using SCIEX TripleTOF 6600 system with the following properties: ion source: electrospray ionization (ESI); scan mode: information-dependent acquisition (IDA); mass range: 50–1000 *m*/*z*; TOF-MS accumulation: 200 ms/spectrum; dynamic background subtraction; and software: Analyst TF 1.7.1 (Sciex, Concord, ON, Canada). Raw data were processed through Metware Cloud platform aat https://cloud.metware.cn (accessed on 2 August 2025) for peak alignment and annotation, multivariate statistical analysis (PCA/OPLS-DA), and Kyoto Encyclopedia of Genes and Genomes (KEGG) pathway enrichment.

### 2.9. Statistical Analysis

All data were expressed as mean ± standard error (SEM) and analyzed using SPSS 27.0. Intergroup differences were assessed by Student’s *t*-test for post hoc comparisons. Statistical significance was defined at *p* < 0.05. A statistical trend was defined as 0.05 < *p* < 0.10.

## 3. Results

### 3.1. Effects of Hydrogen-Rich Water on Growth Performance and Diarrhea Incidence in Weaned Piglets

HRW supplementation improved growth performance in weaned piglets. Compared with the control group, ADFI was significantly increased in the H group (*p* = 0.005); ADG showed a tendency to increase (*p* = 0.094); FGR showed no statistical intergroup difference (*p* = 0.632); and diarrhea incidence rate was substantially reduced in HRW piglets (*p* = 0.001) (Table 1). These findings demonstrate that HRW intervention improves ADFI and diarrhea incidence in weaned piglets.

### 3.2. Effects of Hydrogen-Rich Water on Serum Antioxidant Parameters

**Figure 1:**
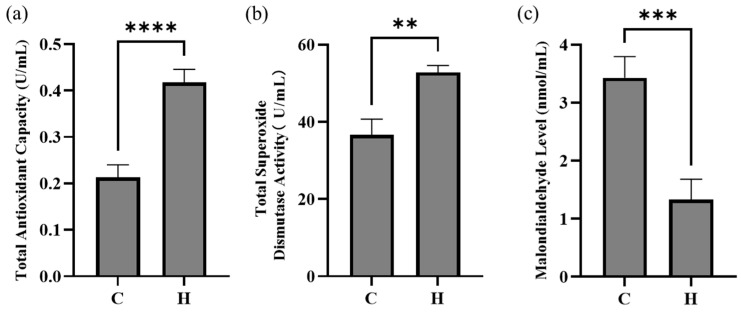
Serum antioxidant profiles in weaned piglets: (**a**): total antioxidant capacity, (**b**): total superoxide dismutase activity, (**c**): malondialdehyde concentration in control and HRW groups. C represents the control group, and H represents the hydrogen-rich water group. ** *p* < 0.01, *** *p* < 0.001, **** *p* < 0.0001 vs. control group.

HRW supplementation significantly enhanced systemic antioxidant capacity in weaned piglets. T-AOC was substantially elevated (*p* < 0.01) (Figure 1a); T-SOD activity showed significant enhancement (*p* < 0.01) (Figure 1b); MDA concentration was significantly reduced (*p* < 0.01) (Figure 1c). These data collectively demonstrate that HRW effectively improves antioxidant defense mechanisms in weaned piglets.

### 3.3. Impacts of Hydrogen-Rich Water on Intestinal Morphology

**Figure 2:**
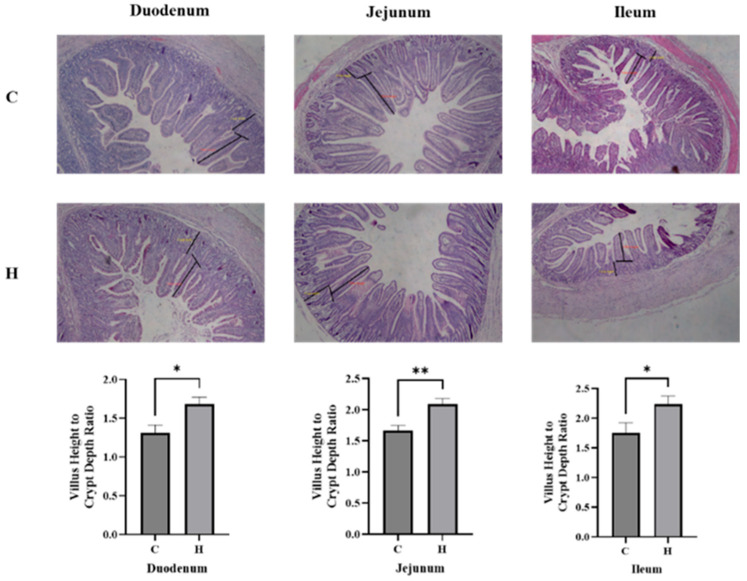
Histomorphometric analysis of small intestinal segments in weaned piglets. C represents the control group, H represents the hydrogen-rich water group, * *p* < 0.05, ** *p* < 0.05 vs. control group.

Given the intimate association between diarrhea pathogenesis and intestinal function, we assessed mucosal architecture alterations (Figure 2). HRW supplementation significantly improved small intestinal morphology in weaned piglets. The VH/CD ratio demonstrated significant increases in duodenum, jejunum, and ileum (Table S1). These morphological improvements demonstrate HRW’s capacity to reinforce intestinal barrier integrity and maintain gastrointestinal homeostasis.

### 3.4. Modulation of Gut Microbiota by Hydrogen-Rich Water

**Figure 3:**
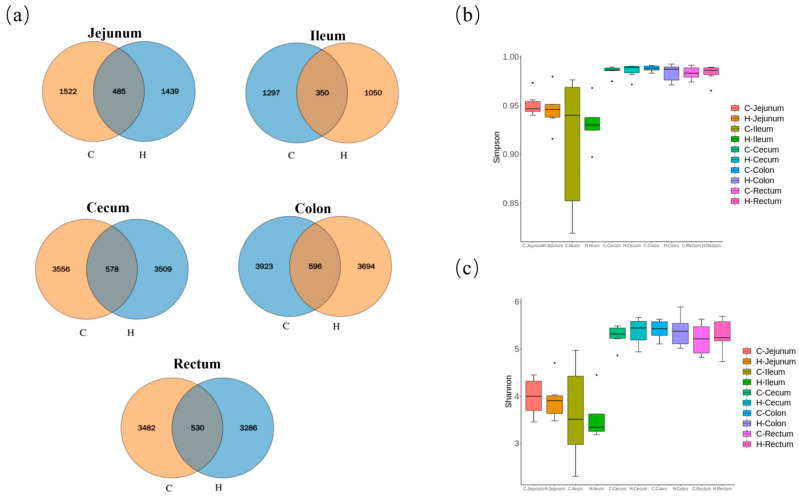
Gut microbial profiling in weaned piglets: (**a**): Venn diagram of core ASVs across intestinal segments, alpha diversity indices (**b**): Simpson, (**c**): Shannon. Note: C represents the control group, and H represents the hydrogen-rich water group. The box plot elements are defined as follows: box limits are upper and lower quartiles, center line is median value, whiskers are minimum and maximum values within 1.5× IQR from the quartiles, and outliers are individual points beyond the whiskers.

The gut microbiota plays a pivotal role in intestinal homeostasis. Microbiological profiling (Table S2) identified 485 amplicon sequence variants (ASVs) in the jejunum, 350 in the ileum, 578 in the cecum, 596 in the colon, and 530 in the rectum (Figure 3a). However, HRW supplementation significantly reduced the abundance of gut ASVs in piglets, potentially attributable to the suppression of detrimental bacteria. Alpha diversity analysis revealed no significant effects of hydrogen-rich water on microbial community diversity (*p* > 0.05 for both Simpson and Shannon indices). Comparative assessment demonstrated higher community diversity in the large intestine (cecum/colon/rectum) relative to the small intestine (jejunum/ileum) (Figure 3b,c).

### 3.5. Gut Microbiota Composition Analysis

**Figure 4:**
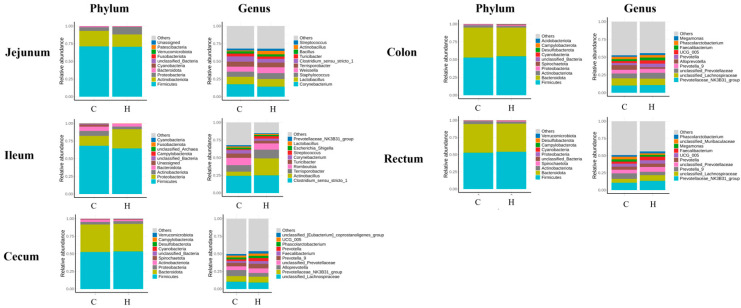
Spatial heterogeneity of gut microbial composition. Note: C represents the control group, and H represents the hydrogen-rich water group. The colored bars show the relative abundance of different colonies.

To investigate the impact of HRW on gut microbiota, a comparative analysis of microbial compositional changes at both phylum and genus levels was conducted across experimental groups. Analysis of intestinal microbial composition demonstrated that, at the phylum level, Firmicutes, Bacteroidetes, Proteobacteria, and Actinobacteriota showed the highest relative abundances in the jejunum, ileum, cecum, and colon, while Firmicutes, Bacteroidetes, Spirochaetes, and Actinobacteriota predominated in the rectum. At the genus level, in the jejunum, high-abundance genera were *Corynebacterium*, *Lactobacillus*, *Staphylococcus*, *Weissella*, *Terrisporobacter*, *Clostridium_sensu_stricto_1* and *Turicibacter*. In the ileum, predominant genera included *Clostridium_sensu_stricto_1*, *Actinobacillus*, *Romboutsia*, *Turicibacter*, *Corynebacterium*, *Streptococcus*, and *Lactobacillus*. In the cecum, major genera consisted of *unclassified_Lachnospiraceae*, *Prevotellaceae_NK3B31_group*, *Alloprevotella*, *unclassified_Prevotellaceae*, *Prevotella_9*, *Faecalibacterium* and *Prevotella*. In the colon, dominant genera were *Prevotellaceae_NK3B31_group*, *unclassified_Lachnospiraceae*, *unclassified_Prevotellaceae*, *Alloprevotella*, *Prevotella_9*, and *Prevotella*. In the rectum, primary genera comprised *Prevotellaceae_NK3B31_group*, *unclassified_Lachnospiraceae*, *Prevotella_9*, *unclassified_Prevotellaceae*, *Prevotella*, *Faecalibacterium*, and *Megamonas* (Figure 4).

### 3.6. LEfSe (Linear Discriminant Analysis Effect Size) Analysis

**Figure 5:**
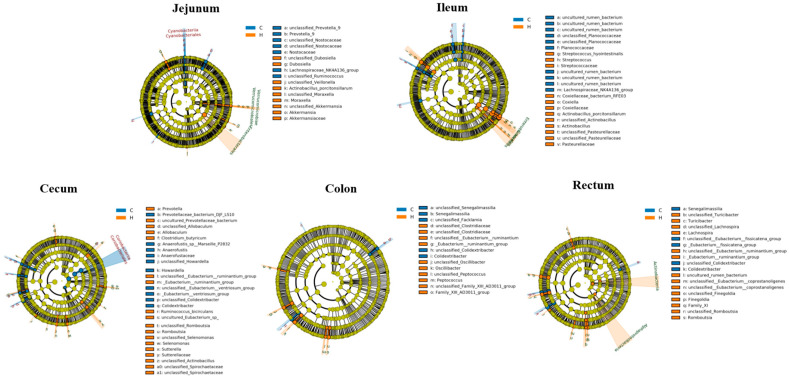
Cladogram of discriminant microbial taxa identified by LEfSe analysis. Note: C represents the control group, and H represents the hydrogen-rich water group. The radial phylogenetic tree illustrates taxonomic hierarchies from phylum to species level (innermost to outermost rings). Each node represents a taxonomic unit at a given level, with node diameter proportional to relative abundance. Taxa showing no significant differences across groups are uniformly colored in yellow. Differentially abundant taxa are colored according to the group where they exhibit highest abundance. Node colors correspond to experimental groups, with each colored node indicating a microbial biomarker significantly enriched in that particular group.

To test the hypothesis that HRW modulates piglet gut homeostasis through microbial community restructuring, LEfSe was employed to analyze microbiota composition and identify signature bacterial taxa across experimental groups. Figure 5 displays the cladogram illustrating differentially abundant microorganisms from phylum to species level across intestinal segments. In the jejunum, *s_unclassified_Akkermansia*, *f_Akkermansiaceae*, *g_Akkermansia*, *s_unclassified_Veillonella*, *c_Verrucomicrobiae* and *g_Dubosiella* were enriched in the H group. In the ileum, *g_Streptococcus* and *f_Pasteurellaceae* showed enrichment in the H group. In the cecum, *g_Prevotella*, *s_Ruminococcus_bicirculans*, *f_Sutterellaceae* and *s_Selenomonas* were enriched in the H group. In the colon, *g_Peptococcus*, *g_Oscillibacter* and *g_Family_XIII_AD3011_group* exhibited enrichment in the H group. In the rectum, *g_Lachnospira*, *g_Romboutsia* and *g_Eubacterium_ruminantium_group* were enriched in the H group.

### 3.7. Prediction Analysis of Microbial Functional Genes

**Figure 6:**
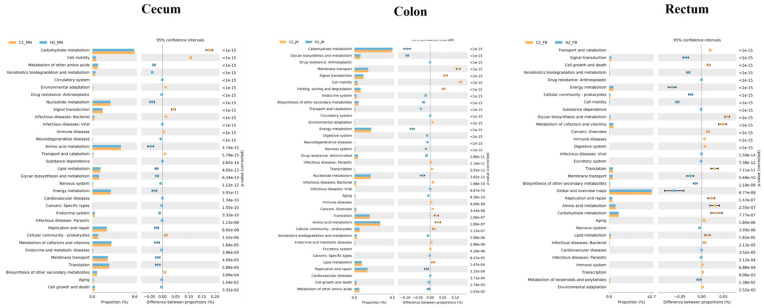
Prediction of microbial community function. Note: C represents the control group, and H represents the hydrogen-rich water group. The left panel displays the relative abundance proportions of functional features across two experimental groups. The central section illustrates the differential abundance proportions with 95% confidence intervals. The rightmost column reports the corresponding *p*-values.

To validate whether HRW modulates piglet intestinal function through microbial-mediated signaling pathways, functional profiling of microbial communities was performed using PICRUSt2. This analysis predicted metagenomic functions based on marker gene sequences, enabling inference of pathway-level alterations. Functional prediction of the microbial communities was performed using PICRUSt2 software by analyzing marker genes within microbial samples. In the cecum, the signature microbial communities in the H group were primarily enriched in functions related to metabolism of other amino acids, xenobiotic biodegradation and metabolism, nucleotide metabolism, amino acid metabolism, and energy metabolism. In the colon, the signature microbial communities in the H group were mainly enriched in functions including carbohydrate metabolism, glycan biosynthesis and metabolism, biosynthesis of other secondary metabolites, transport and catabolism, energy metabolism, nucleotide metabolism, and replication and repair. In the rectum, the signature microbial communities in the H group were predominantly enriched in functions such as signal transduction, xenobiotic biodegradation and metabolism, energy metabolism, cellular community prokaryotes, cell motility, membrane transport, and global and overview maps (Figure 6). These results demonstrate that the core function of the signature microbial communities in the H group involves the regulation of substance metabolism. This indicates that hydrogen-rich water improves intestinal substance metabolism and maintains intestinal homeostasis in piglets through modulating the gut microbiota.

### 3.8. Effects of Hydrogen-Rich Water on Liver Metabolites in Weaned Piglets

**Figure 7:**
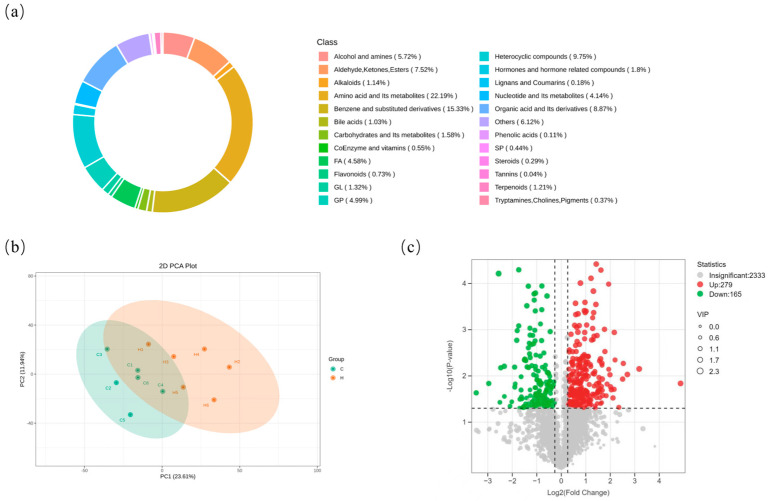
Identification of liver metabolites: (**a**): Composition of metabolite classes. (**b**): Principal component analysis of samples. (**c**): Volcano plot of differentially abundant metabolites. C represents the control group, and H represents the hydrogen-rich water group.

Weaning induced redox imbalance in piglets, leading to hepatic oxidative damage. Metabolite identification in the liver yielded a total of 2333 metabolites (Table S3). These metabolites belonged to the following major classes (Figure 7a): amino acid and its metabolites (22.19%), benzene and substituted derivatives (15.33%),heterocyclic compounds (9.75%),organic acid and its derivatives (8.87%), aldehydes, ketones, esters (7.52%), and alcohols and amines (5.72%). Principal component analysis (PCA) of the samples revealed distinct differences in metabolites between the C group and the H group (Figure 7b). Using the Orthogonal Partial Least Squares-Discriminant Analysis (OPLS-DA) model, metabolites with a Variable Importance in Projection (VIP) score > 1 and a *p*-value < 0.05 were identified as significantly differentially abundant metabolites. The screening results are presented in a volcano plot (Figure 7c). Compared to the C group, the H group exhibited 4444 significantly altered known metabolites. Among these, 279 metabolites were significantly upregulated, and 165 metabolites were significantly downregulated. These results indicate that hydrogen-rich water may regulate the host’s metabolic network in a multi-target and multi-pathway manner.

### 3.9. Analysis of Differentially Abundant Metabolites and KEGG Pathway Enrichment

**Figure 8:**
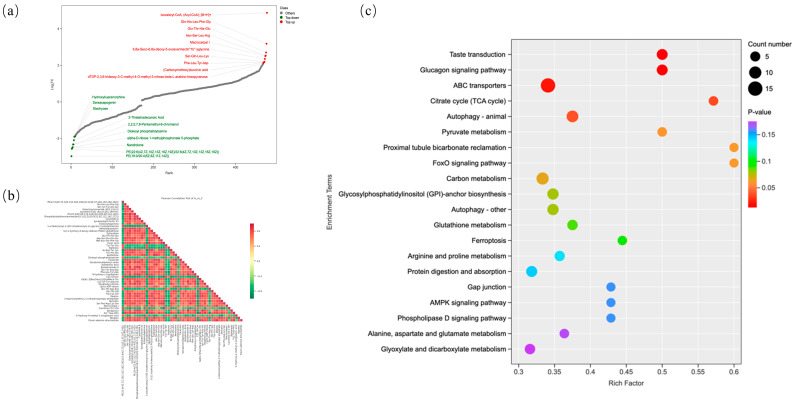
Analysis of differentially abundant metabolites: (**a**): Ranking of differentially abundant metabolites based on fold change. (**b**): Heatmap of correlations among differentially abundant metabolites. (**c**): KEGG pathways enriched by differentially abundant metabolites. Note: (**a**): Each point represents a distinct metabolite. Green points denote the top 10 most significantly downregulated metabolites, while red points indicate the top 10 most significantly upregulated metabolites. (**b**): Color intensity represents the magnitude of Pearson correlation coefficients, with red hues indicating strong positive correlations and green hues denoting strong negative correlations. Darker shades correspond to higher absolute values of the correlation coefficient. (**c**): Point color intensity reflects the significance of enrichment (*p*-value magnitude), with deeper red hues indicating more statistically significant enrichment. Point size corresponds to the number of differentially abundant metabolites enriched in each pathway.

Based on fold-change values, the top 10 significantly upregulated and downregulated metabolites were ranked (Figure 8a). Metabolites significantly elevated in the H group (VIP ≥ 1, *p* < 0.05 vs. control) were Isovaleryl-CoA, Gln-His-Leu-Phe-Gly, Glu-Thr-His-Glu, Asn-Ser-Leu-Arg, Macrocarpal I, 6,8a-Seco-6,8a-deoxy-5-oxoavermectin’1b’aglycone, Ser-Gln-Leu-Lys, Phe-Leu-Tyr-Asp, Carboxymethoxy succinic acid, and dTDP-2,3,6-trideoxy-3-C-methyl-4-O-methyl-3-nitroso-β-L-arabino-hexopyranose. Metabolites significantly reduced in the H group (VIP ≥ 1, *p* < 0.05 vs. control) were PE(18:0/20:4(5Z, 8Z, 11Z, 14Z)), PE(22:6(4Z, 7Z, 10Z, 13Z, 16Z, 19Z)/22:6(4Z, 7Z, 10Z, 13Z, 16Z, 19Z)), Nandrolone, α-D-ribose 1-methylphosphonate 5-phosphate, Dioleoyl phosphatidylserine, 2, 2, 5, 7, 8-Pentamethyl-6-chromanol, 3-Thiatetradecanoic Acid, Stachyose, Sarsasapogenin and Hydroxybuprenorphine. Correlation analysis of the top 50 VIP-ranked metabolites identified the top five positively and negatively correlated pairs (Figure 8b). The positive correlations were Phosphatidylethanolamine(dm18:1(11Z)/20:5(5Z, 8Z, 11Z, 14Z, 17Z)) and Bouillonamide A, Phosphatidylethanolamine(dm18:1(11Z)/20:5(5Z, 8Z, 11Z, 14Z, 17Z)) and Lys-Tyr-Tyr-Arg-Val, Daidzin and Flavin adenine dinucleotide, Bouillonamide A and Simeprevir, Glu-Tyr-Arg-Asp and Ser-Phe-Asp-Lys-Ser. The negative correlations were C22 GlcCer and PLC Thio-PIP2, Kojibiose and Phe-Leu-Tyr-Asp and Kojibiose and Sericetin, PE(22:6(4Z, 7Z, 10Z, 13Z, 16Z, 19Z)/22:6(4Z, 7Z, 10Z, 13Z, 16Z, 19Z)) and Ser-Tyr-Tyr-Gln-Ser, PE(22:6(4Z, 7Z, 10Z, 13Z, 16Z, 19Z)/22:6(4Z, 7Z, 10Z, 13Z, 16Z, 19Z)) and Decabromodiphenyl oxide. Based on the results of the differentially abundant metabolite analysis, KEGG pathway enrichment analysis was performed. As shown in Figure 8c, the key metabolic pathways significantly enriched by the differentially abundant metabolites include citrate cycle (TCA cycle), ATP-binding cassette transporters (ABC) transporters, glucagon signaling pathway, taste transduction, pyruvate metabolism, autophagy, proximal tubule bicarbonate reclamation, Forkhead box O (FoxO) signaling pathway, carbon metabolism, glycosylphosphatidylinositol (GPI)-anchor biosynthesis, glutathione metabolism, ferroptosis, arginine and proline metabolism, protein digestion and absorption, phospholipase D signaling pathway, Adenosine 5‘-monophosphate (AMP)-activated protein kinase (AMPK) signaling pathway, gap junction, alanine, and aspartate and glutamate metabolism. Notably, several of these enriched pathways such as autophagy, FoxO signaling pathway, glutathione metabolism, ferroptosis, and the AMPK signaling pathway are closely associated with oxidative stress responses.

## 4. Discussion

Weaning induces stress in piglets, leading to reduced growth performance, increased diarrhea incidence, and elevated mortality, thereby compromising economic returns [9]. Previous studies have demonstrated that hydrogen-rich water administration mitigates Fusarium mycotoxin-induced reductions in ADG and ADFI in piglets [10]. In the present study, compared to the control group, hydrogen-rich water treatment, significantly decreased diarrhea incidence (*p* < 0.05) and significantly increased ADFI (*p* < 0.05). The absence of body weight gain despite increased ADFI may imply metabolic reprioritization, potentially toward immune modulation or oxidative stress mitigation. Future studies quantifying energy partitioning via calorimetry are warranted to test this hypothesis.

Weaning stress disrupts redox homeostasis in piglets, leading to excessive production of reactive oxygen species (ROS) that induces cellular and tissue damage [11]. This pathophysiological cascade is consistent with reports demonstrating hydrogen-rich water’s efficacy in mitigating oxidative stress across species. Murine models showed significant reduction in MDA and enhanced activities of SOD and GSH-Px [12]. Our findings extend these observations to swine, demonstrating that hydrogen-rich water significantly increased serum T-AOC, enhanced T-SOD activity, reduced serum MDA levels. Collectively, these data indicate that hydrogen-rich water potentiates systemic antioxidant defenses in weaned piglets.

Intestinal morphology and function are critical for health and development in weaned piglets. Newborn piglets exhibit immature intestinal development, and weaning stress induces structural and functional alterations in the intestinal mucosa, leading to villus atrophy, crypt deepening, increased permeability, and impaired digestive absorption and barrier function [1]. The elevated villus height-to-crypt depth (VH/CD) ratio signifies substantial improvement in intestinal functionality, primarily through enhanced absorptive efficiency and optimized epithelial homeostasis [13]. Existing studies demonstrate that hydrogen-rich water consumption significantly increases the VH/CD ratio in the small intestine of piglets fed Fusarium-contaminated diets [10]. In the present study, compared with the control group, hydrogen-rich water significantly elevated the VH/CD ratio, across all three segments of the small intestine. These observations suggest that hydrogen-rich water may ameliorate intestinal damage induced by weaning stress in piglets.

Studies indicate that hydrogen-rich water consumption may improve gut microbiota composition, maintain microecological equilibrium, and promote intestinal health [14]. The present study demonstrated that hydrogen-rich water supplementation exerted no significant effect on gut microbial alpha diversity. However, previous research has established that hydrogen-rich water significantly attenuates intestinal oxidative damage, corrects dysbiosis, reduces pathogenic bacteria, and enriches probiotics in heat-stressed mice [15]. This aligns with reports suggesting that, while hydrogen influences specific microorganisms, its impact on overall community structure remains limited [16], possibly due to complex metabolic interactions within the gut. These collective findings indicate that, while hydrogen-rich water may not alter overall microbial diversity, it potentially optimizes microbiota functionality through restructuring community composition. Our results confirmed higher microbial community diversity in the large intestine than in the small intestine. This aligns with the established literature reporting colonic bacterial densities reaching 10^11^–10^12^ CFU/mL in humans, contrasting with merely 10^3^–10^5^ CFU/mL in the small intestine [17]. Such orders-of-magnitude disparity directly contribute to the observed diversity gap. This phenomenon likely relates to differential transit times; ingested food undergoes prolonged retention in the large intestine, providing an extended fermentation window that enhances microbial colonization and diversity. LEfSe analysis identified 9 significantly discriminant taxa in jejunum, 4 in ileum, 13 in cecum, 8 in colon, and 7 in rectum. In the H group, small intestinal enrichment included *f_Akkermansiaceae*, *g_Akkermansia*, and *s_unclassified_Veillonella*. These taxa regulate intestinal barrier integrity, immune responses, inflammatory cytokine secretion, host metabolic pathways, and short-chain fatty acid metabolism [18,19]. Reduced oxidative stress may favor *Akkermansia* proliferation [20], potentially contributing to decreased diarrhea incidence and improved intestinal morphology. Cecal enrichment featured *s_Ruminococcus_bicirculans* (plant polysaccharide utilization, cellulose/amylase activity) [21] and *s_Selenomonas* (carbohydrate metabolism via glycolysis and TCA cycle) [22]. Colonic enrichment included *Peptococcus* (polysaccharide/protein degradation; acetate/butyrate production). Rectal enrichment comprised *g_Romboutsia* (carbohydrate metabolism, Short-chain fatty acids generation) and *g_Lachnospira* (complex carbohydrate degradation; acetate/propionate/butyrate production) [23]. Collectively, these microbial shifts suggest enhanced fermentation of undigested dietary residues in the large intestine, potentially improving feed utilization efficiency and providing supplemental energy for growth. Hydrogen-rich water may thus alleviate weaning stress, maintain intestinal homeostasis, and promote nutrient absorption in piglets.

Metabolic profiling revealed significant alterations in hepatic metabolites following hydrogen-rich water administration. KEGG pathway enrichment analysis identified significant enrichment in the citrate cycle. This cycle generates Guanosine Triphosphate, reduces Flavin Adenine Dinucleotide and Nicotinamide Adenine Dinucleotide to FADH_2_ and Nicotinamide adenine dinucleotide, and serves as the core energy metabolism hub by providing ATP and metabolic intermediates for cellular growth and function [24]. Other enriched pathways including the glucagon signaling pathway may modulate intestinal function via gut hormone regulation [25]. ABC transporters maintain homeostasis by extruding xenobiotics, toxins, and metabolites [26]. Glutathione metabolism is critical for redox balance, antioxidant defense, and detoxification [27]. Autophagy is essential for redox homeostasis; ROS-activated autophagy mitigates oxidative damage by degrading damaged components [28]. FOXO signaling enhances oxidative stress resistance and mitochondrial integrity [29]. Ferroptosis regulated cell death driven by Glutathione peroxidase 4 inactivation [30]. AMPK/SIRT1 pathway attenuates endoplasmic reticulum stress and mitochondrial dysfunction in oxidative contexts [31]. Collectively, these metabolic reprogramming events indicate that HRW ameliorates weaning stress primarily through modulating oxidative stress pathways.

## 5. Conclusions

This study demonstrates that HRW intake significantly increased ADFI and enhanced serum antioxidant capacity in weaned piglets while concurrently reducing diarrhea incidence. Furthermore, it improved intestinal morphology, modulated gut microbial community composition, and altered hepatic metabolism. These comprehensive improvements were likely mediated through the regulation of oxidative stress pathways.

## Figures and Tables

**Table 1:** Effect of hydrogen-rich water on growth performance and diarrhea rate of weaned piglets.

Growth Performance	C	H	*p*-Value
ADFI (g/d)	377.05 ± 3.04 ^a^	388.23 ± 1.53 ^b^	0.005
ADG (g/d)	239.65 ± 11.50	265.39 ± 14.61	0.094
FGR	1.62 ± 0.10	1.55 ± 0.14	0.632
Diarrhea rate (%)	27.38 ^a^	9.22 ^b^	0.001

Note: C represents the control group, H represents the hydrogen-rich water group. Data are presented as mean ± standard deviation (SD). Within each row, values with different lowercase superscript letters indicate statistically significant differences (*p* < 0.05). Values without superscript letters indicate no significant difference (*p* > 0.05).

## Data Availability

The datasets used or analyzed during the present study are available from the corresponding author upon reasonable request.

## Supplementary Materials

The following supporting information can be downloaded at: https://www.mdpi.com/article/10.3390/biology14080997/s1, Table S1: Intestinal villus height and crypt depth; Table S2: Gut microbiota analysis; Table S3: Liver metabolomics data.

## Abbreviations

The following abbreviations are used in this manuscript:

HRW	Hydrogen-rich water
ADG	Average daily gain
ADFI	Average daily feed intake
FCR	Feed conversion ratio
VH/CD	Villus height-to-crypt depth
